# Structural Resilience Across the Life Course: Perspectives From Older Adults Racialized as Black

**DOI:** 10.1111/jan.70222

**Published:** 2025-09-16

**Authors:** Boeun Kim, Alicia K. Cooke, Tiffany J. Riser, Melissa D. Hladek, Paris B. Adkins‐Jackson, Laura J. Samuel, Roland J. Thorpe, Sarah L. Szanton

**Affiliations:** ^1^ College of Nursing University of Iowa Iowa City Iowa USA; ^2^ School of Nursing Johns Hopkins University Baltimore Maryland USA; ^3^ Mailman School of Public Health Columbia University New York New York USA; ^4^ Bloomberg School of Public Health Johns Hopkins University Baltimore Maryland USA

**Keywords:** community, empowerment, health, qualitative approaches, social determinants of health

## Abstract

**Aim(s):**

This study explored perceptions of older adults racialised as Black on structural resilience across the life course.

**Design:**

A qualitative descriptive study.

**Methods:**

Using purposive sampling, we recruited 15 Black adults aged 50 and older residing in Baltimore, Maryland, including individuals possessing historical or current knowledge of the community. Semi‐structured interviews were conducted to elicit participants' experiences with structural resources during childhood, adulthood and late adulthood. Interviews were audio‐recorded, transcribed verbatim and analysed using content analysis.

**Results:**

Of the 15 participants, three identified as male (20.0%) and 12 as female (80.0%), with an average age of 70.9 ± 8.2 years. The analysis identified nine categories of structural resilience, confirming its multifaceted and dynamic nature. Common categories present across all life stages included: Built environment, civic engagement, food and housing, healthcare, and social capital and cohesion. Life stage–specific categories included child and family services, educational supports, and workforce development supports during childhood and adulthood, and financial support during adulthood and late adulthood.

**Conclusion:**

These categories were interdependent and spanned across life stages, illustrating the dynamic, cumulative and relational qualities of structural resilience. Furthermore, structural resources were identified as key to safeguarding, empowering and restorative responses to adversity.

**Impact:**

These findings contribute to the development of a nuanced, life course–informed framework of structural resilience and highlight the need for ecological strategies that address structural forces shaping health and well‐being, particularly among older adults racialised as Black.

**Reporting Method:**

This study was reported in accordance with the Consolidated Criteria for Reporting Qualitative Research checklist.

**Patient or Public Contribution:**

No patient or public contribution.

## 1 Introduction

Resilience refers to a process of effectively negotiating, adapting to or managing significant sources of stress or trauma (Windle 2011). It involves the use of assets and resources within individuals and their environments to enhance capacity for positive adaptation, recovery and rebound in the face of adversity and risk (Windle 2011; Ungar and Theron 2020; Szanton and Gill 2010). Divergent health trajectories and outcomes can emerge from unique interactions between individuals and their family, community and broader societal structures in response to adversity (Szanton and Gill 2010). Notably, environmental and societal resources that enhance capacity for resilience are not equitably distributed (Thomas Tobin et al. 2021), which limits the effectiveness of these assets in maintaining and promoting health among certain groups and thereby contributing to persistent health disparities. In the United States, Black individuals—more precisely, those *racialized as Black*—have historically faced, and continue to experience, structural racism and health disparities (Phelan and Link 2015). We intentionally use the phrase *racialized as Black* to underscore that race is a social construct imposed by societal systems of power, not an inherent biological trait (Jacob et al. 2023). In this paper, Black refers to a socially constructed category encompassing individuals who self‐identify as having African ancestry, regardless of country of origin. Structural racism, which is embedded in housing, education, employment, earnings, benefits, credit, media, health care and criminal justice systems, has shaped the daily lives of Black communities and produced cumulative adversities over the life course (Bailey et al. 2017). Yet, despite these economic, social and environmental adversities, many older adults *racialized as Black* demonstrate remarkable strength, agency and adaptation (Taylor Jr. et al. 2020), underscoring the importance of examining resilience from their perspective.

Despite growing recognition of the importance of resilience, structural resilience has been unexplored, particularly among older adults racialized as Black. Structural resilience refers to a process of leveraging structural resources at the community and societal levels (Szanton and Gill 2010; Panter‐Brick and Leckman 2013) not only by providing tangible support to recover from, resist, or rebound during or after adversity, but also by functioning proactively to build capacity, strengthen preparedness, and reduce exposure to potential challenges before they arise. Given the repeated, long‐term exposures to structural barriers and their lived experiences navigating a lifetime of racialized adversity, older adults *racialized as Black* offer critical insight into the role of structural resources in fostering resilience. However, much of the existing resilience literature continues to emphasise individual or psychological traits, with limited attention to the broader structural contexts that shape health and well‐being—particularly among older adults racialized as Black. Furthermore, resilience is not a fixed trait; it evolves over the life course as the types and intensity of adversities change with age (Windle 2011), and it can be cultivated and strengthened as individuals confront and overcome challenges throughout the lifespan (Jakovljevic 2017). For example, adversity experienced during childhood—a critical developmental period for stress response systems and emotion regulation (Feder et al. 2019)—may lead to worse outcomes, with the effects of adverse childhood experiences persisting into young adulthood and adulthood, influencing both well‐being and resilience (Haczkewicz et al. 2024; Li et al. 2023). A life course lens allows us to examine how individuals draw upon structural resources not only to respond to adversity at a given point in time, but also to understand how resilience is shaped cumulatively and contextually across different stages of life. To fully grasp the multifaceted nature of resilience (Babić et al. 2020; Gartland et al. 2011), it is essential to move beyond individual‐level constructs and explore it through a structural and life course lens. Therefore, structural resilience demands closer scrutiny.

Although some studies have explored related constructs—like community resilience, social‐ecological resilience and health system resilience (Castleden et al. 2011; Fridell et al. 2020; Patel et al. 2017)—most have centred on environmental or systematic crises, such as natural disasters or public health emergencies. However, there remains a gap in understanding the role of structural resilience in shaping how individuals respond to everyday adversities and challenges, such as structural racism, health events, poverty, or the loss of loved ones. For instance, one study developed a scale to assess perceived community resilience, focusing on a community's capacity to thrive during significant changes like natural disasters (Lindberg and Swearingen 2020). Another study evaluated the capacities and processes required for resilient health systems in response to infectious disease outbreaks and natural hazards (Meyer et al. 2020). Similarly, research on organisational resilience often centres on extreme events such as terrorism, climate change, economic and political crises and disease outbreaks (Barasa et al. 2018). These frameworks, while informative, overlook the more routine but persistent adversities that many older adults racialised as Black face daily. Without a clear and nuanced understanding of structural resilience in this context, the field lacks the tools necessary to assess and ultimately intervene on the structural factors that shape health inequities.

Accordingly, this qualitative study aimed to explore perceived structural resilience over the life course, as described through the lived experiences of older adults racialized as Black. Specifically, the study sought to identify community‐ and societal‐level resources across childhood, adulthood and late adulthood, describing how these resources—both in the face of adversity and through everyday interactions—shape health and well‐being. Investigating structural resilience among older adults racialized as Black through a life course lens offers a critically important opportunity to deepen our theoretical understanding of resilience and inform the development of more effective, culturally responsive interventions.

## 2 Methods

### 2.1 Study Design

This study employed a qualitative descriptive design using content analysis (Sandelowski 2000; Elo and Kyngäs 2008) to comprehensively describe resources and assets at the community and societal levels over the life course that were available to older adults racialized as Black. The content analysis approach was chosen because it is well suited to exploring structural resilience, a relatively unknown phenomenon (Vaismoradi et al. 2013). We applied an inductive approach focused on manifest content, using constant comparison to identify and extract resources and assets from participants' experiences. This study adhered to the Consolidated Criteria for Reporting Qualitative Research (COREQ) reporting guidelines (Tong et al. 2007).

### 2.2 Participants

A purposeful sampling method was used to maximise variation and richness in responses (Campbell et al. 2020), considering access to socioeconomic resources and residential locations. Eligible participants were adults aged 50 and older, racialized as Black, and residing in Baltimore, Maryland. Participants were recruited from lists of participants from prior research projects who had previously expressed interest in participating in future studies, and through snowball sampling. Older adults racialized as Black who were actively involved in advocacy or community support, or who had close knowledge of such activities, were also recruited through existing relationships of non‐author collaborators affiliated with the research team (participants 7, 14 and 15). Baltimore City is characterised by residential segregation, often called ‘the Black Butterfly’, with segregated communities with large frequencies of residents racialized as Black (henceforth Black communities) in the east and west, marked by stark disparities in access to opportunity (Brown 2021). The research team recruited eligible individuals from across the city. Baltimore City was chosen for its historically significant context in examining structural resilience among older adults racialized as Black. The city has long been shaped by entrenched structural racism, yet it also has a rich history of Black‐led community organising, civic engagement, and cultural preservation, making it an ideal setting to explore how structural resources are leveraged to foster resilience over the life course. Participants were recruited until data saturation was reached (Hennink and Kaiser 2022), which occurred at 15. Among 15 participants, three identified as male (20.0%) and 12 as female (80.0%). Excluding one participant who declined to disclose their age, the average age of the remaining 14 participants was 70.9 years (SD = 8.2), ranging from 52 to 80 years.

### 2.3 Data Collection

Based on their preferences and availability, semi‐structured individual interviews were conducted in their homes or other private spaces, such as a reserved meeting room at the interviewer's institution. Participants were guided through a life course interview that explored their experiences with environmental and societal resources across three life stages: Childhood (0–18), adulthood (19–49) and later life (50+). Questions focused on where they lived, who or what supported them during challenges, their involvement in community and social groups, use of public spaces, experiences with social services, and perceptions of neighbourhood cohesion, safety and engagement. To facilitate recall of events over the life course and reduce recall bias, visual aids were utilised, and participants were encouraged to use personal photographs for reminiscence. The interview guides and visual aids are included in the Appendix S1. Interviews ranged in duration from one to one and a half hours. The interviews were audio‐recorded and transcribed verbatim.

### 2.4 Research Team and Reflexivity

The interdisciplinary study team comprised researchers of diverse racial and ethnic backgrounds—including self‐identified Asian, Black, White and Hispanic—and represented a range of ages (20s–50s) and research experience, from students to full professors. The team originated from a larger project aimed at reducing structural discrimination in historically marginalised communities. We acknowledge that our social identities, lived experiences and professional roles may have influenced how we developed the interview guide, engaged with participants and interpreted narratives. At least one team member racialised as Black was involved in each stage of data collection, analysis and interpretation to ensure that lived experience informed both the process and the findings. The entire study process was led by a senior faculty member (SS) with extensive experience in qualitative research and the topic of interest. Interviewers and participants had no pre‐existing relationships, except in cases where individuals involved in advocacy or community support were intentionally recruited to provide in‐depth and comprehensive lived experiences related to structural resources.

### 2.5 Data Analysis

From March 2024 to December 2024, we conducted a content analysis to understand structural resilience across the life course from the lived experiences of older adults racialised as Black. Categories of community‐ and societal‐level resources were derived from participants' narratives, and the roles of these structural resources were also explored, following the preparation, organising and reporting phases to ensure the rigour and trustworthiness of the findings (Elo and Kyngäs 2008; Elo et al. 2014). After repeatedly reviewing the transcripts to gain a comprehensive understanding of the data, two coders (BK and AC) independently conducted open coding of all transcripts and developed an initial codebook through consensus. During the open coding process, the coders met regularly to discuss their interpretations and compare them against the transcript. Then, using the drafted codebook, the two coders separately coded one transcript, checked inter‐coder agreement, and modified the codebook to improve clarity. Once agreement was established, the coders independently coded the remaining transcripts. A third coder (TR) reviewed and confirmed the codes in alignment with the transcripts. The codes were grouped to extract categories through debriefing sessions among the three coders and finalised via whole‐team consensus. Data were managed and analysed using ATLAS.ti software, version 25 (ATLAS.ti Scientific Software Development GmbH).

### 2.6 Ethical Considerations

This study was approved by the Institutional Review Boards at Johns Hopkins University, Baltimore, Maryland (No. IRB00206642, approved on March 25, 2021). All participants provided informed consent prior to participation. All research procedures followed the approved study protocol.

## 3 Results

**FIGURE 1:**
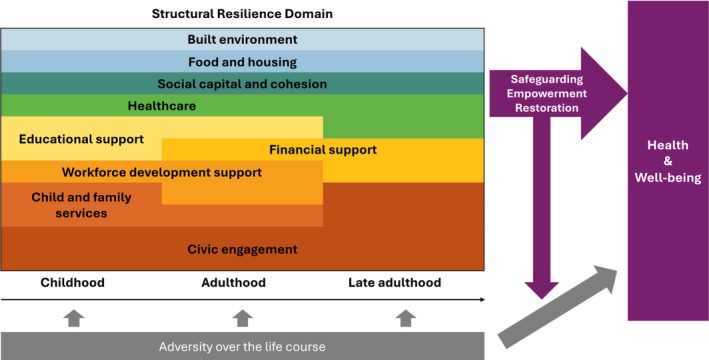
Conceptual framework of structural resilience over the life course. Structural resilience consisted of nine key categories: (1) built environment, (2) child and family services, (3) civic engagement, (4) educational supports, (5) workforce development supports, (6) financial support, (7) food and housing, (8) healthcare and (9) social capital and cohesion. These categories were interconnected within and across life stages. The relative importance of each category is represented by its width in the figure. Each category contributed to health and well‐being through one or more of three mechanisms: safeguarding (mitigating exposure to adversity), empowerment (strengthening capacity to respond to adversity), and restoration (supporting recovery from adversity), depending on the type and timing of adversity encountered.

Eight categories emerged from participants' experiences during childhood (0–18 years), nine categories were identified for the adulthood period (19–49 years), and six categories were extracted for late adulthood (50 years and older). We also identified three roles of structural resilience: (1) safeguarding, by mitigating or preventing exposure to adversity; (2) empowering, by enhancing the capacity to adapt before facing adversity and preparing for future challenges and (3) restorative, by providing tangible support to recover from adversity (Figure 1).

### 3.1 Childhood (Ages 0–18)

Participants experienced a range of severe childhood adversities, including experiences of racial discrimination, bullying, physical and sexual abuse, poverty and living in systemically disadvantaged neighbourhoods that lack consistent access to essential services and opportunities. Structural resources across eight categories supported efforts to maintain and promote well‐being during childhood: (1) built environment, (2) child and family services, (3) civic engagement, (4) educational supports, (5) workforce development supports, (6) food and housing, (7) healthcare and (8) social capital and cohesion.

*Built environment*—including recreational amenities, grocery stores, spaces for intellectual activities, facilities promoting physical activity and accessible transportation—provided opportunities for diverse activities and essential resources. These features empowered participants and helped prepare them to manage and adapt to adversity by promoting physical, mental and social well‐being, reflecting an empowering role of structural resilience. For example, Participant 11 recalled: ‘They always had a movie theater. … went to the movies, and it was nice’. Participant 10 described how the built environment encouraged physical activity and social connection within the neighbourhood: ‘The gym was at the school. We would go play games, or we had plays and stuff. … a lot of neighborhood kids would come; neighborhood adults would come’. Transportation also played a meaningful role in expanding access to community resources. Participant 4 recalled: ‘Back then you would pay like a fee, and you can ride the bus like all day. … So, we would catch the bus like all day going around like to downtown’.

*Child and family service*—including foster care and social services—provided stability and critical assistance when participants or their families were in need, particularly when they could not be cared for by their biological parents, illustrating the restorative role of structural resilience. Participant 2 shared a memory: ‘They used to take us on trips… We had a nice time. I loved my foster parents’.

Additionally, church activities and community involvement served as important sources of community support (*civic engagement*), demonstrating the empowering role of structural resilience. Participant 15 remembered organised civic activities that brought neighbours together:

They used to have this program called Clean Block program, operation champ, and you would clean up your neighborhood and the cleanest neighborhood will get a prize… You are just so proud to clean up your block to win something. I think everybody was a winner.

Educational resources played a significant role in shaping participants' lives, exemplifying empowerment and restorative roles of structural resilience. Access to specialised and high‐quality education, extracurricular programs, and dedicated teachers contributed to their development and helped them overcome academic challenges (*educational supports*). As Participant 1 reflected on the transformative support of a teacher: ‘At first, I felt bad because I felt like I was dumb… My teacher… taught me a lot because she came to love me and my family… she would take time to help me… She helped me all the way’. Participant 14 also recalled: ‘The principal was marvelous. She tried to bring everybody in and to learn’.

*Workforce development supports*—such as part‐time jobs and job training programs—played a valuable role in preparing participants for employment, extending resources and strengthening capacity to withstand adversity (empowerment role of structural resilience). Participants 4 also recalled: ‘It was called School to Work or something, some kind of program they had… So, they would teach you like job training and send you on job interviews’.

Some participants lived in publicly supported housing, and many reported food sharing across communities (*food and housing*), reflecting the restorative role of structural resilience. Participant 12 recalled: ‘When we lived in the housing project, it was a very comfortable place. All of the children, we were close’. Participant 13 described common informal acts of food sharing across communities: ‘I've been there when people just leave a box of vegetables on the front porch, so it was people helping each other to survive’.

*Access to healthcare* was also an essential resource for participants during childhood, supported by proximity to clinics and availability of health insurance, reflecting the restorative role of structural resilience. Participant 11 recalled a neighbourhood clinic that provided accessible care: ‘It was connected to the housing projects. They had the well‐baby clinic which was a two‐room clinic, one for the mothers and the children, and one room for the doctor’. Participant 1 emphasised the importance of insurance coverage in accessing needed care: ‘When I was young… I couldn't hear, so then the city told me that my hand just cost me $2500 a piece, and that's when I had Blue Cross/Blue Shield, and they paid for it’.

Lastly, *social capital and cohesion* provided a sense of safety, mutual support, and community belonging—played a safeguarding role by enhancing neighbourhood safety, fostering watchful and supportive relationships, and facilitating community involvement. Participant 3 reflected on the influence of a supportive neighbourhood: ‘The entire neighborhood was a village. I lived around a lot of schoolteachers… So, they had a big influence on me’. Participant 2 described a safe neighbourhood environment: ‘It was clean. They didn't do killing back then … Everybody was getting along’. As Participant 9 shared memories of close‐knit communal life:

Back in the day, we were a real close‐knit neighborhood. If you cried, we cried, so we got into a lot of cooking… We would come out and sit outside, and we would laugh and joke and watch TV or listen to music and things like that.

### 3.2 Adulthood (Ages 19–49)

Participants faced numerous hardships in adulthood, including structural racism, living in systemically disadvantaged neighbourhoods, experiences of violence, workplace injuries, job loss, financial difficulties, health issues (e.g., chronic diseases), separation, the loss of loved ones and challenges related to parenting and caregiving. Structural resources available in adulthood were identified across nine categories: (1) built environment, (2) child and family services, (3) civic engagement, (4) educational supports, (5) workforce development supports, (6) financial support, (7) food and housing, (8) healthcare and (9) social capital and cohesion. While most categories were also present during childhood, financial support newly emerged as a distinct category during adulthood.

*Financial support* helped participants make necessary home modifications and cope with financial hardship, demonstrating the restorative role of structural resilience. Government‐funded programs were instrumental in improving home accessibility. Participant 4 recalled: ‘When that Medicare waiver program kicked in, it helped a lot because they put a ramp on the house. They put a chair lift in the house. They made sure everything was handicap accessible, like changing the bars in the bathroom. So, it really, really helped’. Participant 8 shared their experience receiving income support during a time of instability: ‘When I got here, I had to get public assistance because my daughter and I… didn't have any income. My two sons, when I first got here, they had SSI’.

As in childhood, *built environment* in adulthood continued to offer spaces and resources that supported recreation, physical activity and intellectual engagement. Participant 14 described how access to a library fostered learning: ‘I got my kids, … we loved school every day, with me and reading with them and taking them to the library. We used to go to the library and bring bags of books home, and I liked that’.

Childcare in a neighbourhood, caregiver support from federal programs, and social services facilitated by social workers helped alleviate challenges related to caregiving and parenting (*child and family services*), exemplifying the restorative role of structural resilience. Participant 4 reflected on the impact of a government‐supported caregiving program: ‘Once that program (i.e., Medicare waiver program) kicked in, it was a huge blessing to us, huge, huge blessing because they pay somebody to come in and care for her’. Participant 13 also shared how access to nearby childcare supported employment: ‘When I came back, he was 2 years old, so I put him in a daycare because I had to get a job, but there was a daycare near me’. Participant 6 recalled how a social worker provided social services: ‘They had social workers in the school. I felt like I could talk to her when things were going on with my family… She was white, but she was really concerned about everybody. … no matter who you were’.

Participants also described receiving or providing advocacy and support through community organisations, professional associations and social groups (*civic engagement*) as a meaningful structural resource that served both safeguarding and empowerment roles. Participant 7 shared their experience of supporting a lesbian, gay, bisexual, transgender and queer (LGBTQ) organisation:

Actually, being an activist… It started out as the Gay Alliance, sort of white and male dominated, but some lesbians and some lesbians of color and me and other individuals that were cofounding and involved… and I really felt comfortable there.

Participant 14 discussed their involvement in a professional association that fostered mentorship: ‘So, it started from a few pharmacists in Baltimore to a statewide expansion of a society of Black pharmacists that… mentor younger Black pharmacists or people starting out in the health space’.

Participant 2 reflected on grassroots efforts led by mothers advocating against community violence:

Mothers got together… Because a lot of times when their children are victimized by crime, usually grounds of retaliation if it's a police officer. These mothers are so hurting but they're trying to send a clear message, stop the shooting, reduce the violence.

*Educational supports* (e.g., scholarships, work‐study programs, affordable tuition) enabled participants to pursue higher education and secure better positions in adulthood, reflecting the empowering role of structural resilience. Participant 12 reflected:

I earned my first master's degree. There was federal money that paid for my tuition, so I did that…. So they paid for our tuition, and we got a $500 stipend, as I recall. Not just me, but anybody who was in the program.

Participant 4 described how earning a degree expanded their opportunities:

… So, then I ended up going back to school there and getting my degree… So, I started as an admin there and once I graduated, … my director at the time was really supportive and, … he made sure that he changed my role a little bit, gave me a promotion.

Career advancement support—such as promotion pathways, job training, and networking opportunities like career fairs—helped participants secure employment or attain better positions (*workforce development supports*), reflecting the empowering and restorative roles of structural resilience. Participant 15 described how members of the Black firefighter community supported one another in the face of structural barriers:

My dad and others in the Black community, they were not getting recognized, and they weren't getting promoted like others were. So, they just formed together, they would do study groups together to make sure they would score high on a test, doing everything they could possibly do so they wouldn't get passed up the promotion. Sometimes it worked, sometimes it didn't.

Participant 14 shared how job training programs helped women who had experienced intimate partner violence gain skills and secure employment:

They've never been to a place where they've been working. And they lose boyfriends and their husbands because they don't want them to have more money than them… There was a lot that we put them through training that they learned a skill… To get a higher level of training that they could work in different places.

Public or community‐based food and housing assistant programs, such as food stamps and public housing projects, were available (*food and housing*), illustrating the restorative role of structural resilience. Participant 9 described the food assistance program: ‘We got food stamps and things like that, assistance, and that was the big thing back then to make it’. Participant 4 shared their involvement in a church‐based outreach that provided food and clothing to those in need: ‘I was a part of their outreach ministry, … we would go out on the street, and we would pray for people and then tell them to come around to the church because we would serve food …. there was a food pantry. If they needed clothes, they could get clothes’.

*Access to healthcare services* helped participants in recovering from physical and mental health crises, while hospice care services helped alleviate their caregiving challenges (the restorative role of structural resilience). Participant 4, who was grieving the loss of a loved one, described how access to mental health services through their employer provided support during a difficult time: ‘The grief counselor? … We have the EAP program, the Employee Assistance Program. So, I called through there, through that referral line and they connected me with someone’. Participant 6 shared their experience with limited, but helpful hospice support while caring for a loved one at home: ‘I had to take care of him and finally had to get hospice, but hospice came to the house, but the thing of it, they only came like 4 h during the week’.

*Social capital and cohesion* in adulthood were reflected through strong community ties, mutual support, and the presence of Black‐owned businesses or organisations that provided essential resources (safeguarding and restorative roles of structural resilience). Black‐owned businesses, such as supermarkets and salons, served as more than just commercial establishments—they became spaces for connection, support and advocacy. Participant 14 described how Black‐owned supermarkets evolved into a broader network of community resources:

It was Black owned supermarkets that then were expanded. It's Charlie Brown Super Pride Movement that focused on safe and accessible supermarkets, and then he expanded it into his co‐interest, which was safe and affordable healthcare, and so it was eventually five Black owned and operated medical centers across Baltimore from about 1965 to the late 90's, each anchored by a Black pharmacist.

Participant 15 recalled the mutual support that developed between Black firefighters and the Black community:

Then eventually things started to change, as more and more people in the Black community supported the Black firefighters, and vice versa. It just helped with a lot of challenges in the neighborhood. They would do Thanksgiving, they would get things, buy stuff for the community, food and everything they could to help. You can always stop past the firehouse and get things that you need. … Whatever, they would take donations, and they also would give people what they need.

Similarly, Black‐owned media outlets, such as *The Afro newspaper*, played a crucial role in fostering social cohesion by amplifying community voices and preserving cultural identity.

The Afro was the best. That was the only real thing we had to read that was just for us, and would tell our stories. So yeah. Afro was one of the best things that happened back then. … Afro, we could read a lot of stories and feel like we could connect with other home families. (Participant 15)

### 3.3 Late Adulthood (Ages 50+)

Participants reflected on a lifetime of resilience having navigated adversities and hardships throughout both childhood and adulthood. In late adulthood, they continued to face challenges such as struggling to pay medical bills, coping with serious health conditions—including HIV and other chronic diseases—and experiencing the loss of loved ones. Structural resources in late adulthood were available across six categories: (1) built environment, (2) civic engagement, (3) financial support, (4) food and housing, (5) healthcare and (6) social capital and cohesion.

Similar to earlier life stages, *built environment* in late adulthood—including commercial and retail spaces, facilities that promote physical activity, recreational amenities and religious institutions—continued to offer important opportunities for social engagement and participation in diverse religious and community activities (empowering role of structural resilience). Participant 12 shared how a local church became a space for connection and involvement: ‘It's a Methodist Church, and I've been going there’.

In late adulthood, participants became resources themselves by actively joining and contributing to community organisations or social groups (*civic engagement*), reflecting the empowering role of structural resilience. Participant 13 shared their advocacy activities:

And I started going to conferences… I would always volunteer… So, I participated in a campaign with Baltimore City Health Department called ‘Positively’… they did which was to educate people on getting care.

Participant 11 described their involvement in community‐based efforts to improve neighbourhood:

I attend church at East Baltimore. And then I also belong to the Family Health Centers of Baltimore. I'm on the board there… And back then they were trying to get things straightened out so that it wouldn't be as bad. There was a gang there, and they were wiped out, … people wanted Cherry Hill to be better, and they started forming committees and other things to help it, get it off the ground.

*Financial support*—including assistance with medical services, prescription costs, and home modifications—helped participants manage their health and physical conditions (the restorative role of structural resilience). Participant 13 shared their experience receiving financial assistance after losing coverage for their HIV medication:

Fortunately, I have a case manager… So, I called my case manager… She found out what had happened… And she gave me a voucher, like $150, which gave me five pills. But she managed to find a foundation that gave me a grant for $7,500. That's good for 1 year. And so, I was able to pay for my meds for the past 2 months using the grant money.

Participant 9 described how access to affordable medical services and home modification programs supported their health and well‐being: ‘Capable, they came out. They gave me a brand new; I have a brand new bed. I have a new toilet seat which keeps me from being so low’.

Everything is either from some program or something that I've been on that covers everything, what Medicare or Medicaid don't cover, so my medicine gets pretty well taken care of. I have very expensive medicine, but Hopkins takes care of me.

Community food assistance programs and housing navigation support provided food and helped them find stable housing (*food and housing*), demonstrating the restorative role of structural resilience. Participant 11 described receiving food support: ‘Oh, they have food giveaways, and then during the pandemic, I was getting all these boxes’. Participant 10 shared how a community‐based program helped them secure housing: ‘So at STAR. We have different counselors there. We have different people down there that help you find places and information’.

Furthermore, participants emphasised that having health insurance, access to hospice care services, and receiving quality care were essential for accessing *healthcare* in late adulthood (the restorative role of structural resilience). Participant 12 noted the role of insurance coverage in maintaining access to care: ‘So, I have insurance as well as Medicare. And so that's, they cover that’. Participant 9 shared their experience receiving attentive, patient‐centered care: ‘Johns Hopkins, they make you think you, make me think that I'm the only patient they have. Through my doctor, nurses and all, they make me feel like I'm the only patient and I'm like I know that I'm not’.

Lastly, participants received—or provided—social support through structured support groups and informal support networks (*social capital and cohesion*), illustrating the safeguarding and empowering roles of structural resilience. These networks played a crucial role in promoting emotional well‐being and fostering a sense of community. Participant 10 shared the value of being part of a support group: ‘I have one support group… That's our women's support group. That's for people who have HIV’. Participant 10 also described the importance of social networks within their residential community:

But there are a lot of people in this building that don't have the support that I have. I get phone calls when they don't see me. They knock on the door when they don't see me. But I do a lot. I try to do a lot for the elderly, because some people don't have family.

## 4 Discussion

This qualitative study explored structural resilience across the life course from the perspectives of older adults racialized as Black and identified structural resilience factors across nine key categories: (1) built environment, (2) child and family services, (3) civic engagement, (4) educational supports, (5) workforce development supports, (6) financial support, (7) food and housing, (8) healthcare and (9) social capital and cohesion. These categories played a pivotal role in building and fostering resilience throughout the life course in the context of a wide range of severe adversities. While these categories collectively shaped resilience, their relevance varied by life stage. The categories were also deeply interconnected—both within and across stages of life—highlighting the dynamic and multifaceted nature of structural resilience. The nine key categories of structural resilience across the life course and their roles in promoting health and well‐being were presented in Figure 1. Importantly, this study also found evidence that older adults become resources themselves over the life course by contributing to their communities to support others facing adversity. This work makes a critical and timely contribution to resilience research by illuminating the structural resources that shape coping and adaptation in the face of adversity across the life course, operating not only as protective factors but also as evolving sources of strength.

Participants in this study experienced a wide range of serious and extreme adversities across their lives. Despite profound challenges, participants survived, adapted, and ultimately thrived into late adulthood, maintaining their well‐being. Even though we identified a comprehensive range of structural resources that supported resilience, participants primarily emphasised intrapersonal and interpersonal resources, such as faith and family, as key sources of strength. In our interviews, much of the discussion centered on these interpersonal and intrapersonal resources as ways of overcoming challenges; however, these narratives are not presented in detail here, as they fall outside the scope of this study. This pattern may reflect findings from existing literature suggesting that adults racialized as Black often perceive the responsibility for overcoming adversity as falling primarily on the individual. This perception is deeply rooted in American values that emphasise self‐reliance, strength, and spirituality, particularly among women racialized as Black (Woods‐Giscombe et al. 2023; Abrams et al. 2014). Alternatively, this tendency may also reflect the enduring effects of structural racism, whereby economic and sociopolitical resources are systematically withheld from or inequitably distributed within Black communities. Such inequities—shaped by historical and ongoing discriminatory policies, residential segregation, and unequal power relations—have constrained opportunities and limited access to supportive resources across the life span (Dennis et al. 2025; Szanton et al. 2010), reinforcing patterns in which individuals must rely heavily on personal and community strengths to navigate adversity. Nevertheless, structural resources played a critical role. Even when not always explicitly recognised by participants, the presence of structural supports was essential for overcoming adversity and fostering resilience, particularly among those unable to rely solely on themselves or their immediate networks. These findings underscore the urgent need to ensure the availability of structural resources for Black communities in need.

The narratives of participants revealed structural resilience as a multifaceted and dynamic process. This study identified nine key categories that constitute structural resilience. The nine categories were identified through an inductive analysis of participant narratives; however, the analysis process was guided by our definition of structural resilience, which was informed by the Society‐to‐Cell resilience framework. This Society‐to‐Cell resilience framework conceptualises resilience as a process shaped by the cumulative interaction of factors across six levels: cellular, physiological, individual, family, community and societal (Szanton and Gill 2010). This study focused specifically on community‐ and societal‐level factors that facilitate resilience and, in turn, promote health and well‐being. Furthermore, this study found that the relevance of these categories and the type of support they offered shifted according to participants' needs and exposures over the life course. During childhood, structural resilience was fostered through a range of supports—such as education, community resources (e.g., housing, food), institutional programmes (e.g., foster care), and safe environments for social and physical development (e.g., social cohesion, parks, recreational facilities). During adulthood, structural supports expanded to include continued education, employment opportunities (e.g., job training, professional networks), civic engagement through advocacy and resource provision, support for Black‐owned businesses and culturally specific media, and participation in community‐based resources. Financial support became increasingly important—particularly during young adulthood—and newly emerged as a critical category in adulthood, as participants assumed greater responsibilities for themselves and their families. This included managing household expenses, securing stable housing, accessing food and healthcare, and navigating periods of job instability. In late adulthood, resilience continued to expand through civic engagement and participation in social groups. Financial support and access to healthcare remained important resources for maintaining well‐being.

Importantly, the findings suggest that structural resilience is both cumulative and relational—building over time through the accumulation of supportive resources—and relational, shaped by interactions among resources across the categories of structural resilience. The life course perspective informed the approach by framing structural resilience as a process that develops and operates across multiple stages of life, with past exposures and resources influencing later outcomes (Pearlin et al. 2005). For example, early exposure to supportive environments (e.g., education, nurturing teachers, supportive neighbours) enhanced participants' later capacity to access essential resources—such as employment opportunities, health insurance and other benefits—that were critical for maintaining well‐being and overcoming challenges across the life course. Given the profound impacts of childhood adversity on health and well‐being in later life (Suglia et al. 2018; Merrick et al. 2019), structural resources during childhood that mitigate exposure and buffer its harmful effects can lay the foundation for long‐term resilience and health. Moreover, civic engagement was particularly notable, emerging as a cross‐cutting category through which participants both received and generated resilience. In childhood, participants were more often recipients of community care and support; however, as they grew older, they increasingly became sources of resilience themselves—engaging in volunteerism, political activism, and peer‐led support networks, and serving as active agents of change within their communities. This dual role reflects a communal and collective resilience within Black communities, where civic engagement, mutual aid, and grassroots organising serve as vital strategies to resist structural oppression and foster community strength (Reese and Johnson 2022; Sagiv et al. 2022). The central importance of civic engagement is reflected in the proposed framework, where its relative prominence is represented by its width in Figure 1.

Across categories, three mechanisms emerged from participants' narratives through which structural resilience promoted health and well‐being across the life course: (1) safeguarding, (2) empowerment and (3) restoration. These findings align with the buffering hypothesis, which posits that social support enhances well‐being both by directly providing tangible resources and by buffering the negative effects of stress (Cohen and Wills 1985). These mechanisms illustrate the varied ways in which structural conditions shaped participants' ability to navigate adversity and maintain or regain stability and well‐being. It is important to note that these categories are not mutually exclusive; many operated across multiple mechanisms and life stages. The first mechanism, safeguarding, involves structural resources that prevent or reduce exposure to adversity by creating safe and supportive environments. Categories such as social capital and cohesion and civic engagement played this role by enhancing neighbourhood safety and facilitating community involvement—factors that helped minimise risk and prevent harm. The second mechanism, empowerment, includes resources that build and enhance participants' abilities to manage and adapt to adversity through daily interactions, prior to encountering challenges. This role recognises that resilience can develop not only in response to extraordinary challenges but also through the accumulation of everyday interactions with structural resources (Masten 2001). The built environment, educational supports, and workforce development resources contributed to this process by providing access to resources and spaces and opportunities for physical activity, intellectual development and social engagement. These categories build knowledge, skills, and networks needed to overcome structural barriers and achieve long‐term stability. In doing so, they strengthened individuals' sense of agency and preparedness for future challenges. The third mechanism, restoration, encompasses structural resources that provide tangible support during or after adverse events, helping individuals recover and regain stability following hardship. Healthcare access, food and housing security, financial support, and child and family services were particularly vital in helping participants stabilise during and after hardship, manage chronic health conditions, meet basic needs, and fulfil caregiving responsibilities—including instances where alternative caregiving arrangements were necessary during childhood. These resources directly addressed the consequences of adversity and enabled individuals to recover, adapt and rebound towards well‐being.

This study has several limitations, and its findings should be interpreted with caution. The sample is geographically limited to Baltimore City and may not fully capture the diversity of structural resources encountered by older adults racialized as Black in other regions of the United States. As such, the transferability of the findings may be limited. Further studies in varied geographic and sociopolitical contexts will be critical to exploring both common and unique pathways of resilience across communities. In addition, the study focused exclusively on older adults racialized as Black; future research should include more racially and ethnically diverse groups to examine how structural resilience may manifest similarly or differently across different populations.

## 5 Conclusion

This study contributes to a growing understanding of how structural resilience is shaped across the life course. It advances the development of a nuanced framework that highlights the multifaceted, dynamic, cumulative, and relational natures of structural resilience and their safeguarding, empowering, and restorative roles against adversities among older adults racialized as Black across the life course. While these findings do not offer definitive conclusions, they provide a valuable foundation for continued inquiry and dialogue on the structural dimensions of resilience. The study underscores the need to move beyond individual‐level approaches and towards broader, ecological, and life course–informed strategies that account for the structural forces influencing health and well‐being. An essential component of nursing is recognising individuals as part of dynamic systems in constant interaction with their environment (Szanton and Gill 2010). Identifying structural resources that enable positive adaptation and thriving despite adversity and understanding their roles in promoting health and well‐being can inform practice and policy advocacy aimed at addressing structural determinants of health to promote well‐being and reduce inequities.

## Author Contributions

All authors have agreed on the final version and meet at least one of the following criteria: (1) substantial contributions to conception and design, acquisition of data, or analysis and interpretation of data; and (2) drafting the article or revising it critically for important intellectual content.

## Ethics Statement

This study was reviewed and approved by the Institutional Review Board at Johns Hopkins University (No. IRB00206642). All participant provided informed consent prior to participation.

## Conflicts of Interest

The authors declare no conflicts of interest.

## Supporting information


**Appendix S1:** jan70222‐sup‐0001‐AppendixS1.docx.

## Data Availability

The data supporting this study are not publicly available due to confidentiality and privacy considerations. A portion of the de‐identified data may be made available upon reasonable request and with appropriate institutional approvals.
