# Supplementary material for: Structural Resilience Across the Life Course: Perspectives From Older Adults Racialized as Black
Source: J Adv Nurs. 2025 Sep 16;82(4):3575–85. doi: 10.1111/jan.70222 (PMC12994672; doi:10.1111/jan.70222)
Supplement: Supplementary file 1 — Appendix S1: jan70222‐sup‐0001‐AppendixS1.docx. [file JAN-82-3575-s001.docx]

**Title: Structural Resilience across the Life Course: Perspectives from Older Black Adults**

**Resilience Interview Guide**

*For each question, follow up about people, places, social structures, programs involved in each story that may hint at “resilience structures.”*

- *Education – HEADSTART, social workers*
- *After school/Camps - church camp*
- *Recreation - Operation CHAMP*
- *Neighborhood - cleanup clubs (Clean Block Campaign), Youth development clubs*
- *Entertainment & Media – The Afro, theater*
- *Advocacy & Civics – mayor programs, involvement in campaigns*
- *Skills or job training programs*
- *Housing  - home purchase incentive program*
- *Neighborhood groups – block captains*
- *Social services for food, income, education, or child care support*

I am going to ask you questions from three different time periods of your life, ages 0-18, 18-50, and over 50.

Please share any objects, momentos, or photos if they align with questions I ask. If not, we can speak about them at the end. You may choose to skip any questions you don’t want to answer.

There are no right or wrong answers. We are trying to interview all different kinds of people as part of this project. We won’t judge anything you say.

**Part A. From ages ~0-18:**

- Where did you live during this time?
- Describe a memory about how a resource, person, or group of people made you feel supported or helped you overcome a challenge in your schooling?
- What about in your teenage years? Who or what did you feel you could turn to for help? Have you or your family received any assistance (social services or social supports) for lunch, grocery, renting home, or health care?

*[If individuals are mentioned, ask what structures those individuals had as support]*

- What community groups or social groups were you a part of? Or would you have wanted to be a part of? For those you were a part of, what three words would you use to describe how your participation in these groups made you feel?
- Where did you play? (parks, rec centers, pools) – {how often, describe a memory}
- Where did you feel most comfortable or made you feel free? (sexual, religious)

*[If places are mentioned, ask how to get there (transportation)]*

- What are particular activities and events that brought your community or groups you were a part of together?
- Were you proud of the neighborhood you lived in? Did you feel that it was close-knit? Were people willing to help their neighbors? Was there a willingness to intervene in situations to protect common good?

**Part B. From ages ~19-50:**

- Where did you live during this time?

*[Ask whether a participant received any help for renting or buying a house (e.g., home purchase assistance program, affordable housing)*

- Describe an important moment or turning point that shaped your professional/work trajectory. Have you received any assistance/social service (e.g., child care voucher, food stamps, tuition support) that helped you to overcome any challenges to achieve the professional/work goals?
- What community groups or social groups were you a part of or would you have wanted to be a part of? What are particular activities and events that bring these communities together?
- Who/what did you feel like you could turn to for help?

*[If individuals are mentioned, ask what structures those individuals had as support]*

- Did you utilize public space? What types? Where did your kids play? (parks, rec centers) – {how often, describe a memory}
- Where did you feel free to express yourself? Were there any places you liked to visit in your neighborhood? – {how to get there, felt safe, describe a place}
- What do you feel helped your children/grandchildren succeed?
- What did you share with others?
- Describe an important relationship event (engagement, wedding, birth of child or family member) – who or what helped you through the experience?
- Describe an experience that made you feel suddenly more mature/older/grown up.
- Were you proud of the neighborhood you lived in? Did you feel safe to walk/play/exercise in your neighborhood? Did you feel that it was close-knit? Were people willing to help their neighbors? Was there a willingness to intervene in situations to protect common good?
- Did you feel engaged in community issues and that your community’s voice was heard? Describe an example.

**Part C. From age 50 until now:**

- Describe any major life transitions (move, illness, death of a loved one, new caregiving responsibilities, family milestones) – who or what helped you through them? Have you received any assistance/social service (e.g., caregiving service, food stamps, grocery, renting or buying home, affordable housing) that helped you to overcome any challenges that you might have during the life transition periods?

*[If illness mentioned, ask about health insurance and paying out of pocket medical expense. If caregiving responsibility mentioned, ask about any assistance received (e.g., Medicare supported nursing homes).*

- What community groups or social groups are you a part of or would you want to be a part of? {What are particular activities and events that bring these communities together?}
- Did you utilize public space? What types? (parks, rec centers) – {how often, describe a memory} Are there any places you liked to visit in your neighborhood? – {how to get there, felt safe, describe a place}
- Who/what do you feel like you could turn to for help?

*[If individuals are mentioned, ask what structures those individuals had as support]*

- Are you proud of the neighborhood you live in? Do you feel safe to walk/play/exercise in your neighborhood? Where do you go for grocery shopping? How to get there? Did you feel that it is close-knit? Are people willing to help their neighbors? Is there a willingness to intervene in situations to protect common good?
- Do you feel knowledgeable about resources in community and that they were available for you to access? Describe an example
- Did you feel engaged in community issues and that your community’s voice was heard? Describe an example.

Did we discuss any objects, momentos, or photos you had thought about from the questions asked during the prep call?

May I take a picture of the objects, momentos, and/or photos you showed me?

End interview

**Visual Aids**

*Please use the provided visual aids to help participants recall events and experiences across their life course.*

**Childhood**


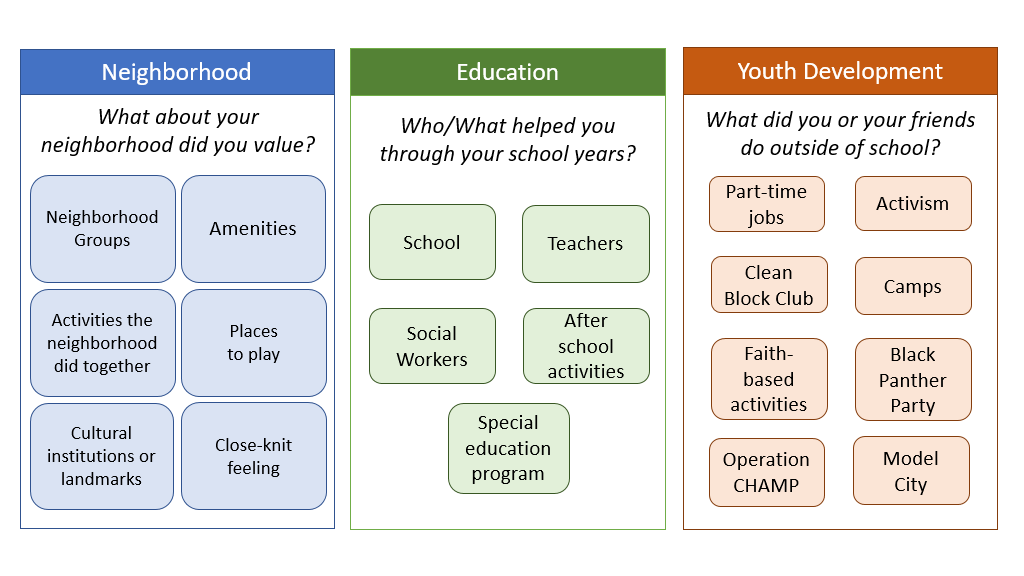


**Adulthood**
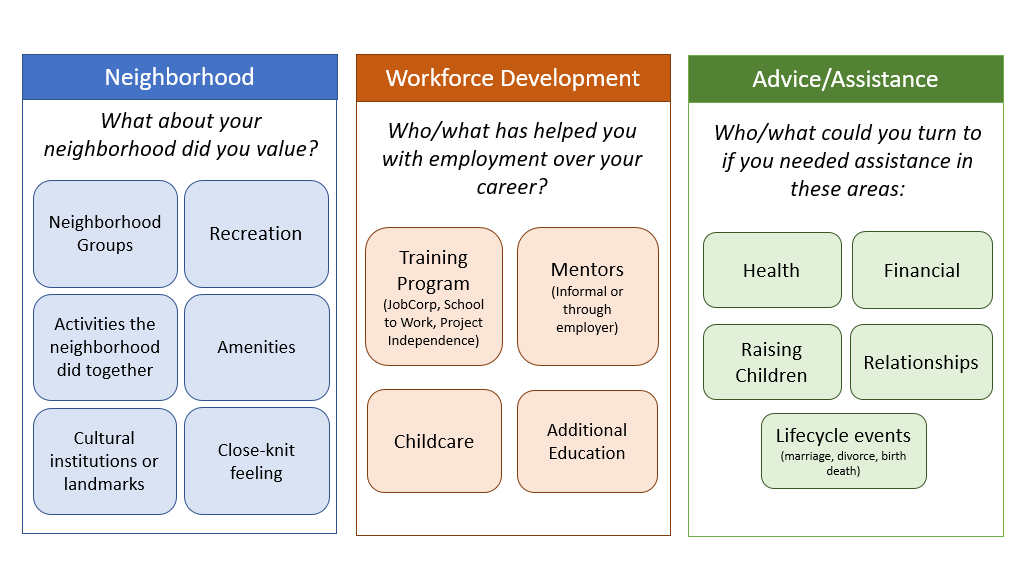
**Late adulthood**
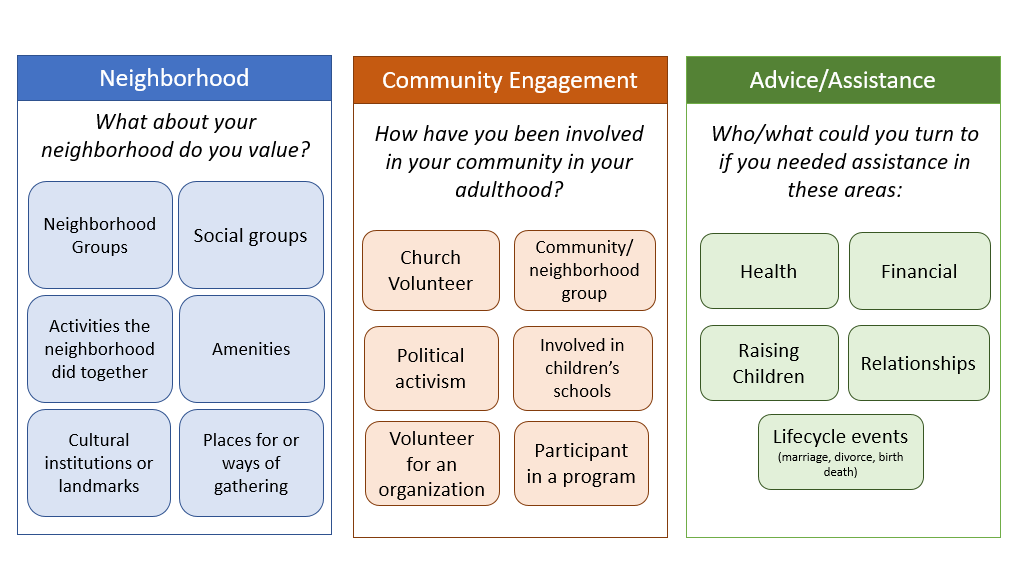


Note. Historical photographs of Black communities are not included here due to copyright restrictions.
